# Modelling the 200 m Front-Crawl Performance Predictors at the Winter Season Peak

**DOI:** 10.3390/ijerph17062126

**Published:** 2020-03-23

**Authors:** Mário J. Costa, Catarina C. Santos, Daniel A. Marinho, António J. Silva, Tiago M. Barbosa

**Affiliations:** 1 Department of Sport Sciences, Polytechnic Institute of Guarda, 6300-559 Guarda, Portugal; 2 Research Center in Sport Sciences, Health Sciences and Human Development, CIDESD, 5001-801 Vila Real, Portugal; marinho.d@gmail.com (D.A.M.); ajsilva@utad.pt (A.J.S.); barbosa@ipb.pt (T.M.B.); 3 Department of Sport Sciences, University of Beira Interior, 6201-001 Covilhã, Portugal; catarina.costa.santos@ubi.pt; 4 Department of Sport, Exercise and Health Sciences, University of Trás-os-Montes e Alto Douro, 5001-801 Vila Real, Portugal; 5 Department of Sport Sciences, Polytechnic Institute of Bragança, 5300-252 Bragança, Portugal

**Keywords:** physiology, biomechanics, performance, swimming, training season

## Abstract

This study aimed to identify potential predictors of 200 m front crawl performance at the winter season peak based on the anthropometric, physiological and biomechanical domains. Twelve expert male swimmers completed an incremental 7 × 200 m step test immediately after their most important winter competitions. Measurements were made of: (i) height, body mass and arm span as anthropometrical parameters; (ii) velocity at a 4 mmol·L^−1^ lactate concentration (V4), maximal oxygen uptake (VO_2máx_) and energy cost (C), as physiological parameters; (iii) stroke frequency (SF), stroke length (SL), stroke index (SI) and propelling efficiency (η_p_) as biomechanical indicators; and (iv) 200 m front crawl race time in official long course competitions. Spearman correlation coefficients identified V4 as the single factor having significant relationship with performance. Simple regression analysis determined V4, SI and arm span as the most relevant variables of each group. Multiple linear regression models showed that physiological factors explained better (59%) the variation in performance at this stage of the season, followed by the biomechanical (14%) ones. Therefore, V4 can be one important aspect for training control and diagnosis for those who want to achieve success in the 200 m front crawl at the winter season peak.

## 1. Introduction

The evaluation of the performance determinants is a key element for coaches and sportsmen. Nowadays, at top-level, in individual, closed and cyclic sports such as swimming, races can be won by hundredths of a second. The 200 m front crawl race is one of the most exciting events of the Olympic calendar. It can be a challenge for researchers, coaches and athletes, since it requires deep analysis and hard training in the several energetic pathways to reach the best performance outcome. Thus, insights about how determinant factors affect each race stroke and distance are eagerly needed. 

In last few years, leading research groups have dedicated their attention to having a deeper understanding about the performance-determining factors in both young and adult swimmers. Bivariate analyses were carried out, establishing relationships between a single determinant domain (notably physiology or biomechanics) and performance. The anaerobic threshold [1,2,3], energy cost [4,5] or time-limit and minimum velocity to achieve the maximal oxygen uptake [6,7] were reported as strongly related to swimming performance. Furthermore, it was determined that those physiological factors seem to depend on biomechanics, such as the stroke kinematics [8], the speed fluctuation [9] and the arm’s propelling efficiency [10,11]. 

Another approach was to design exploratory and confirmatory models to establish hierarchical relationships between several determinant domains and performance. This was a step further, providing a deeper understanding about the interplay between several performance determinants and how swimming is a multi-factorial phenomenon. Ribeiro et al. [12] found that 79% of 400 m front crawl performance variations were explained by the velocity at 85% maximal oxygen uptake and velocity with a 4 mmol·L^−1^ lactate concentration. Obert et al. [13] pointed out the maximal oxygen uptake as the single factor determining the 200 and 400 m front crawl performance in adult swimmers. Costa et al. [14] used hierarchical linear modelling and reported that performance improvements (0.11, 1.21 and 0.36 s) throughout two consecutive seasons were mainly due to the duration to reaching a blood lactate concentration of 4 mmol·L^−1^, the stroke frequency and the stroke length variations. Nonetheless, Zacca et al. [15] reported that technical factors in age group swimmers had the highest contribution (71%) to the changes in 400 m front crawl performance over a competitive season, followed by bioenergetics (17%) and anthropometrics (12%).

It is known that training programs, which are well planned with appropriate physiological and technical stimuli, are an effective way to disturb homeostasis and adapt the organism to subsequent improvements. There should be a relationship between external and internal training load; i.e., the training volume, intensity and frequency are components to be used by coaches to elicit different energetic pathways through the season according to the swimmers’ specializations. Swimming season is most of the time split into a winter and a summer season. Swimmers must build shape at least for the major competitions of these two seasons. Most literature reports peak form at the end of the summer season [14]. However, the periodization process is slightly different on the road for the winter or summer peak shape. Commonly, while the first months aim to develop the aerobic energetic band, the last months are spent most on anaerobic and technical training. Therefore, what remain unanswered are the main determinant factors to being at the top level in major winter competitions. The performance variations can be explained by different determinant factors based on different seasonal periods.

The aim of this study was to identify potential predictors of 200 m front crawl performance at the winter season’s peak based on the anthropometric, physiological and biomechanical domains. It was hypothesized that physiological factors would better explain the performance variation, followed by the biomechanics and anthropometrics. 

## 2. Materials and Methods 

### 2.1. Participants 

Twelve expert male swimmers (20.0 ± 3.54 years old; 10.1 ± 3.41 years of training experience; 1.81 ± 0.07 m of height; 73.20 ± 5.33 kg of body mass; 1.87 ± 0.07 m of arm span; 115.03 ± 3.97 s of personal record in the 200 m long course front crawl event) were recruited to participate in this study. The mean personal time represented 700 FINA (International Swimming Federation) points. One subject was an Olympic swimmer, two had participated at World Swimming Championships and one participated in LEN Multinations Junior Meet representing his National Swimming squad. Collectively, the other half of the subjects (*n* = 8) was Portuguese top-20 ranked in the 200 m freestyle event. The swimmers were experiencing nine sessions per week involving low, medium and high aerobic tasks; intense sprint work; and technical drills. Weekly training volume averaged 44 ± 7 km wk^−1^. Swimmers gave their written informed consent before participation in the study. All procedures were in accordance to the Helsinki Declaration regarding human research and were approved by the local ethics board. 

### 2.2. Design and Procedures

Swimmers were tested in the peak shape period of the winter season immediately after major competitions (i.e., Winter National Championships and the Clubs National Championships). On the day before data collection, the swimmers completed a low intensity training session to avoid data bias due to fatigue. The swimmers were tested in the morning, starting with the anthropometric measurements. They were advised to maintain their normal nutritional routines while trying to avoid caffeine ingestion. Warm-up procedures were standardized before each test, featuring continuous swimming at low–moderate intensity and a few drills no longer than 1 km. After that, an intermittent set of 7 × 200 m front crawling, with increasing velocity as described elsewhere [16] for physiological and biomechanical assessments, was used. The velocities increased by 0.05 m·s^−1^ so that swimmers would attain their maximal performance on the last step. Underwater pacemaker lights (GBK-Pacer, GBK Electronics, Aveiro, Portugal), on the bottom of a 50 m swimming pool, were used to control the swimming velocity and to help the swimmers keep an even pace along each lap and step. A 30 s resting period was used between steps to collect blood samples and oxygen uptake measurements for further energetic analysis. Elapsed time for each trial was measured with a stopwatch and to control the swimmer’s pace. Biomechanical measurements were made at the middle of each lap and then used for further calculations.

### 2.3. Measures

Swimmers were only wearing a textile swimsuit and a cap during all anthropometric tests. Height, body mass and arm span were selected as anthropometric variables. Height (in cm) was measured with the swimmer in the anthropometric position, by measuring the distance from the vertex to the floor with a digital stadiometer (SECA, 242, Hamburg, Germany). Body mass (in kg) was measured with a digital weight scale (SECA, 884, Hamburg, Germany). For the arm span measurement (in cm) the subjects stood in an upright position with arms and fingers fully extended and in abduction at 90°. The arm span was considered the distance between the third finger of each hand and measured with a flexible anthropometric measuring tape (RossCraft, Canada).

To determine the velocity corresponding to blood lactate concentration at 4 mmol·L^−1^ (V4), capillary blood samples were collected from the ear lobe to obtain the lactate concentrations with an auto-analyzer (YSI 1500 l, Yellow Springs, OH, USA). Blood collection happened during the 30 s resting periods between steps, and in the 3rd, 5th and 7th min after the end of the protocol. The individual V4 (in m·s^−1^) was calculated by interpolation of the average lactate value (4 mmol·L^−1^), with the exponential curve of lactate/velocity.

Absolute oxygen uptake (VO_2_) was measured immediately after each trial with a portable gas analyzer (Cortex, Model MetaLyzer 3B, Leipzig, Germany). Swimmers were instructed to take their last breaths immediately before touching the wall. After finishing the trial, the swimmer leaned on the wall, while an operator fixed a portable mask for land-based locomotion on his face during the entire recovery. Swimmers were only allowed to start breathing after the masks were put on their faces. The time gap for this transition and the first breathing cycle never exceeded 3 s. The VO_2_ (mL·kg^−1^·min^−1^) reached during each step of the protocol was estimated using the backward extrapolation of the O_2_ recovery curve [17]. The maximal oxygen uptake (VO_2max_) was considered to be the mean value in the 6 s after the VO_2_ detection during the recovery period [17]. The first measure of VO_2_ values before the highest VO_2_ measurement was not considered, which corresponded to the device adaptation to the sudden change of respiratory cycles and of O_2_ uptake. The device adaptation never exceeded 2 s. Other backward extrapolation methods were already been reported [18,19] and have been shown valid for the VO_2max_ measurement. 

The total energy expenditure (E_tot_) was assessed for further inclusion in the energy cost calculation. The E_tot_ was calculated in the last 200 m step, corresponding to the swimmer’s maximal effort [20]:

(1) $$E_{\mathrm{tot}}={\;\mathrm{VO}}_{2}\mathrm{net}+2.7\;\cdot \left[{\mathrm{La}}^{-}\right]\mathrm{net}$$

where VO_2net_ (l kg^−1^·min^−1^) is the difference between the oxygen uptake measured and the oxygen at rest; 2.7 is the energy equivalent (mL O_2_·mmol^−1^·kg^−1^) for lactate accumulation in blood; and La_net_ represents the difference between the lactate measured and the lactate at rest.

Energy cost (C) was calculated based on previous methods [21] and converted into the SI units according to the suggestion of Minetti [22] that 1 mL O_2_ is equivalent to 20.1 J:

(2) $$C=\frac{E_{\mathrm{tot}}}{v}\;$$

Stroke kinematics was measured in all 50 m lap and averaged for each 200 m stage. The *v* was obtained from the distance and 50 m split times. The SF was measured with a crono-frequency meter (Golfinho Sports MC 815, Aveiro, Portugal) from three consecutive stroke cycles, in the middle of each lap. Then, SF values were converted to SI units (i.e., Hz). The SL was estimated as [23]:

(3) $$\mathrm{SL}=\frac{v}{\mathrm{SF}}$$

where SL is the stroke length (in m), *v* is the swimming velocity (in m·s^−1^) and the SF is the stroke frequency (in Hz). The SI is considered as one of the swimming stroke efficiency indexes and was computed as [24]:

(4) SI=v · SL 

where SI is the stroke index (in m^2^·c^−1^·s^−1^), *v* is the swimming velocity (in m·s^−1^) and the SL is the stroke length (in m). The propelling efficiency (η_p_, %) was also estimated as [10]:

(5) $$\eta_{p}=\left(\;\frac{v\;\cdot \;0.9}{2\pi \;\cdot \;SF\;\cdot \;l}\;\right)\;\cdot \;\frac{2}{\pi }$$

where *v* is the swimming velocity (in m·s^−1^), the SF is the stroke frequency (in Hz) and *l* is the arm’s length (in m). The *l* was computed by trigonometrically measuring the arm’s length and considering the average elbow angles during the insweep of the arm pull, as reported by Zamparo [25]. This is considered an approximation of the Froude efficiency. 

Swimming performance (T_200m_) was assessed based on times lists of the 200 m front crawl event during official long course competitions. The time gaps between the race times and the testing sessions did not exceeded more than two weeks.

### 2.4. Statistical Analysis

The normality and homogeneity assumptions were checked with the Shapiro–Wilk and Levene tests, respectively. For descriptive analysis, mean plus one standard deviation, maximum and minimum were computed as central tendency and dispersion measures. 

Ranking Spearman correlation coefficients (r_s_) were calculated to assess the association between performance and remaining variables (*p* < 0.05). Linear regression models were computed based on performance and remaining variables (i.e., anthropometrics, physiology or biomechanics) (*p* < 0.05). Additionally, multiple linear regression models using the backward stepwise procedure were developed by entering all variables (*p* < 0.05). For the determination of the independent variables that most predict the T_200m_, we included the factors that corresponded the necessary procedures to enter into the model. The variables entered in the equation were those with the highest r^2^ from each group and if F ≥ 2.0.

## 3. Results

Descriptive data of anthropometric, physiologic and biomechanical variables are shown in Table 1 along with the Spearman correlation coefficients between T_200m_ and all variables of interest. None of the anthropometric variables showed a significant association with T_200m_ (height: r_s_ = −0.43; *p* = 0.29; body mass: r_s_ = −0.33; *p* = 0.42; arm span: r_s_ = −0.38; *p* = 0.35). Regarding energetic variables, only V4 showed a significant association with T_200m_ (r_s_ = −0.81; *p* = 0.01). Remaining energetic parameters were not associated with T_200m_ (VO_2max_: r_s_ = −0.50; *p* = 0.21; C: r_s_ = 0.02; *p* = 0.95). Similar findings were verified for the biomechanical variables (SF: r_s_ = −0.55; *p* = 0.15; SL: r_s_ = −0.61; *p* = 0.11; η_p_: r_s_ = −0.07; *p* = 0.87; SI: r_s_ = −0.67; *p* = 0.07). 

The relationships between T_200m_ and remaining variables based on linear regression models are shown on Table 2. Simple linear regression models suggested that arm span (r^2^ = 0.26; *p* = 0.20), V4 (r^2^ = 0.59; *p* = 0.03) and SI (r^2^ = 0.48; *p* = 0.06) were the best isolated predictors of T_200m_. Therefore, only these three variables were entered in the final multiple regression model. 

The multiple linear regression model (Table 3) for the T_200m_ explained 73% of the variance. The highest contribution was from the V4 (59%), followed by the SI (14%). Hence, it seems that the physiological variables are the ones that best characterized the 200 m performance variation in that specific stage of the season. 

## 4. Discussion

The main findings were that V4 and SI were the factors that explained best the performance in the 200 m freestyle event. The physiological factors explained (i.e., V4 = 59%) the variance in 200 m front crawl performance of expert male swimmers at this stage of the season best, followed by the biomechanical ones (i.e., SI = 14%).

Data of the present study demonstrated that none of the anthropometric variables showed a significant relationship with T_200m_. Those are measures often used in the identification and development processes of talented performers [26]. Talent ID is a selection process that supposedly culminates with a homogeneous sample of subjects in regard to a few anthropometric features. Upon reaching adulthood, the contributions of anthropometrical traits for performance are not so obvious due to: (i) the end of growth and maturation; (ii) most swimmers having very even morphometric characteristics. For young adult swimmers, other factors may play a more determinant role. Indeed, the main characteristics of our sample, as reported in the methods section, suggest that inter-subject variability is low–moderate. Despite that, the arm span was the anthropometric variable with the highest contribution for performance. 

V4 mean values (1.43 ± 0.05 m·s^−1^) were within the range of what was reported by others for swimmers of similar competitive level [1,27]. The V4 showed a little different between subjects, which may be explained by the better capacity that some swimmers demonstrate to maximize their energy input. Considering the combination of stroke aspects, the less skilled swimmers experience more difficulties in sustaining an aerobic effort [6]. The V4 was the single physiological variable that showed a significant association with T_200m_ (r_s_ = −0.81; *p* = 0.01). Previous papers reported the V4 as a good predictor (r^2^ = 0.79) of middle-distance races, such as 200 m and 400 m freestyle [12]. It is suggested that maximal swimming bouts that take between 2 and 3 min (such as a 200 m freestyle race of sub-elite and elite swimmers) will require the contributions of both aerobic (approximately 60%) and anaerobic systems (approximately 40%) [28]. Each system is best suited to provide a higher contribution to ATP re-synthesis according to the race distance. Despite the aerobic capacity being the main energetic pathway, surprisingly, the VO_2max_ did not present a significant association with the T_200m_. There is a solid body of knowledge stating that aerobic sources (i.e., aerobic power and aerobic capacity) help to build-up endurance fitness [29]. The aerobic capacity can be defined as the energy base system that supports endurance training. The aerobic power is considered the ability to effectively use that energy base system. Aerobic power is determined by VO_2max_. Since the 200 m distance lasts less than 3–4 min, elite swimmers need to have a large energy base system (aerobic capacity) and deliver that content as fast as they can (aerobic power). Probably, at this stage of the season, the 200 m success relies mostly on the aerobic capacity and less on the aerobic power. Indeed, velocities under the intensity of the VO_2max_ have explained the final swimming performances of adult swimmers [12]. In contrast, the VO_2max_ has been found to be significantly correlated with 100 m speed (r = 0.79) in adult swimmers [29]. More insights about these relationships between internal training load (i.e., V4 vs. VO_2_) and external training load (periodization model for winter season) would be gathered with a multiple linear regression modeling.

The present investigation showed that biomechanics did not correlate with T_200m_ at least at this stage of the season. However, the SI was the single parameter showing values near statistical significance (r_s_ = -0.67; *p* = 0.07). Previous papers have reported a high association between SI and energy expenditure [24] and performance [30] in sub-elite and elite swimmers. High SF values are reached based on muscle power and strength. The inability to produce high muscle power in such conditions leads subjects to increase SL in order to maintain swimming velocity. Based on *v* and SL relationship, swimmers improve overall swimming efficiency in terms of SI. Moreover, SI is expected to decrease from longer to shorter events [31]. Probably, SI is a determinant factor in 200 m race distances but not in shorter ones, mostly, at adult age. 

Multiple linear regression models showed that V4 and SI explained 73% of the performance variance. From the two variables included in the model, the V4 contributed with 59% and the SI with 14%. The higher contribution verified of V4 was probably due to the higher percentage of workout focused on the aerobic capacity earlier in the season. Aerobic capacity build-up is one of the first goals in the season [32]. The aim is to promote a solid aerobic base to elicit other energetic bands (e.g., aerobic power and lactate tolerance) later on [1], and to promote fast and effective recoveries between training sets and even races during competitions. Early in the season, the muscle is more sensitive and able to improve the ability to produce energy aerobically [27]. Low-intensity workouts promote a better vascularization of the muscles (increasing aerobic range) by opening blood vessels as demand for oxygen increases. Simultaneously, the total mitochondrial volume increases and facilitates aerobic energy production. The greatest changes in the earlier months of the season are coupled with training volume. Ryan et al. [33] observed that an increase from 32,000 to 49,000 m·wk^−1^ in the first two months induced an improvement in the V4 from 1.21 to 1.39 yds/s in a group of female college swimmers. A couple of papers followed the same trend in elite male swimmers [14,19]. The muscles heighten their glycogen and fat storing capabilities and increase the length of time for which they can perform work [34]. However, due to several years/a decade of systematic and hard training, the margin for further aerobic improvements from a specific point of the season is quite small in such of subjects. Ryan et al. [33] verified that V4 remained between 1.39 and 1.42 m·s^−1^ from the mid until end of the season, despite increases in training volume to an average of 66.000 m·wk^−1^. This suggests that 200 m front crawl specialists should look for further improvements by developing other determinant aspects of performance. Indeed, the periodization process is designed in order to build up a progressive shape through the season. Training in other energetic bands (e.g., aerobic power and anaerobic lactic/alactic) can change the energetic contribution for the final 200 m event. Each swimmer used the most freely chosen energetic combination (e.g., an increased aerobic capacity and lower anaerobic one, or vice versa) to maintain performance at a higher level. The incrementation of technical sets may also change the stroke mechanics relationship, and define the swimming velocity in a more economical way.

Some limitations associated with this study should be noted: (i) being a cross-sectional study yet not considering the baseline data for comparison; (ii) a small sample size; and (iii) not including other measures (i.e., strength and conditioning, motor control or psychological) in the model. 

## 5. Conclusions 

Physiological factors best explained the variation in the performance of T_200m_, followed by the biomechanical ones. Coaches should use regular testing and monitoring of V4 status. This should be done in parallel with the biomechanical assessments. Finally, the training sets should be built for the development of the aerobic energetic pathway for those who want to achieve success in the 200 m front crawl at the winter season peak. 

## Figures and Tables

**Table 1:** Descriptive values of the anthropometrical, physiological and biomechanical variables, and their correlations with 200 m front crawl performance.

Variable	Mean (±1 SD)	Max	Min	Correlation with T_200m_
T_200m_ (s)	117.94 ± 4.88	111.42	120.70	---
Height (m)	1.81 ± 0.07	1.91	1.71	−0.43 (*p* = 0.29)
Body mass (kg)	73.20 ± 5.33	80.10	66.2	−0.33 (*p* = 0.42)
Arm span (m)	1.87 ± 0.07	2.00	1.80	−0.38 (*p* = 0.35)
V4 (m·s^−1^)	1.43 ± 0.05	1.50	1.35	−0.81 (*p* = 0.01)
VO_2max_ (ml·kg^−1^·min^−1^)	70.61 ± 6.44	78.82	63.34	−0.50 (*p* = 0.21)
C (J·kg^−1^·m^−1^)	15.26 ± 1.18	16.85	13.81	0.02 (*p* = 0.95)
SF (Hz)	0.69 ± 0.04	0.73	0.60	−0.55 (*p* = 0.15)
SL (m)	2.48 ± 0.14	2.56	2.37	−0.61 (*p* = 0.11)
SI (m^2^·s^−1^)	4.22 ± 0.31	4.48	3.77	−0.67 (*p* = 0.07)
η_p_ (%)	39.06 ± 1.55	41.43	36.13	0.07 (*p* = 0.87)

η_p_, propelling efficiency; max, maximum; min, minimum; C, energy cost; SF, stroke frequency; SI, stroke index; SL, stroke length; T_200m_, 200 m front crawl time; V4, velocity at 4 mmol·L^−1^ of blood lactate concentration; VO_2max_, maximum oxygen uptake.

**Table 2:** Linear regression analysis to assess the potential relationships between T_200m_ and each group.

Group	Variable	r^2^	Adjusted r^2^	T	*p*	Beta	F	*p*
Anthropometrics	Height	0.22	0.09	3.84	0.01	−0.464	(1;6 ) = 1.65	0.25
	Body mass	0.20	0.08	6.03	<0.01	−0.448	(1;6) = 1.50	0.27
	Arm span	0.26	0.14	3.86	<0.01	−0.510	(1;6) = 2.11	0.20
Physiology	V4	0.59	0.52	5.99	<0.01	−0.769	(1;6) = 8.68	0.03
	VO_2max_	0.25	0.13	7.62	<0.01	−0.500	(1;6) = 1.99	0.21
	C	0.01	−0.16	4.82	<0.01	−0.090	(1;6) = 0.05	0.83
Biomechanics	SF	0.23	0.10	5.28	<0.01	−0.481	(1;6) = 1.80	0.23
	SL	0.04	−0.12	3.86	<0.01	−0.198	(1;6) = 0.25	0.64
	SI	0.48	0.39	8.39	<0.01	−0.691	(1;6) = 5.47	0.06
	η_p_	0.02	−0.14	2.77	0.03	−0.156	(1;6) = 0.15	0.71

η_p_, propelling efficiency; C, energy cost; SF, stroke frequency; SI, stroke index; SL, stroke length; T_200m_, 200 m front crawl time; V4, velocity at 4 mmol·L^−1^ of blood lactate concentration; VO_2max_, maximum oxygen uptake.

**Table 3:** Summary of the model, included in the forward step-by-step regression equation, for predictors of T_200m_.

	Variable	r^2^	Adjusted r^2^	T	*p*	Beta	F	*p*
T_200m_	V4 SI Arm span	0.59 0.73 0.74	0.52 0.63 0.54	5.99 6.71 5.55	<0.01 <0.01 <0.01	−0.769 −0.571 −0.082	(1;7) = 8.68 (2;7) = 6.84 (3;7) = 3.74	0.03 0.04 0.12

SI, stroke index; T_200m_, 200 m front crawl time; V4, velocity at a 4 mmol·L^−1^ of blood lactate concentration.
